# Early retinal synaptic dysfunction and proteomic remodeling precede neurodegeneration in a Parkinson’s disease model

**DOI:** 10.1038/s41531-026-01261-7

**Published:** 2026-01-15

**Authors:** Chae-Eun Moon, Seung Jae Lee, Haesol Shin, Hongkyung Kim, Jun-Ki Lee, Hyunjin Kim, Hyunseung Kang, In Hee Moon, Sung Soo Kim, Hyung Keun Lee, Kyoung Yul Seo, Sung-Rae Cho, Yong Woo Ji

**Affiliations:** 1 Institute of Vision Research, Department of Ophthalmology, Yonsei University College of Medicine, Seoul, Republic of Korea; 2 Department of Ophthalmology, Aerospace Medical Center, Republic of Korea Air Force, Cheongju, Korea; 3 Korea Mouse Sensory Phenotyping Center (KMSPC), Yonsei University College of Medicine, Seoul, Republic of Korea; 4 Department of Ophthalmology, Dongsuwon General Hospital, Suwon, Republic of Korea; 5 Department of Ophthalmology, Yongin Severance Hospital, Yonsei University College of Medicine, Yongin, Republic of Korea; 6 Department of Ophthalmology, Gangnam Severance Hospital, Yonsei University College of Medicine, Seoul, Republic of Korea; 7 Department of Rehabilitation Medicine, Graduate School of Medical Science, Brain Korea 21 Project, Yonsei University College of Medicine, Seoul, Republic of Korea; 8 Research Institute of Rehabilitation Medicine, Yonsei University College of Medicine, Seoul, Republic of Korea; 9 Department of Biohealth Engineering, Graduate School of Transdisciplinary Health Sciences, Yonsei University, Seoul, Republic of Korea; 10 Brain Research Institute, Yonsei University College of Medicine, Seoul, Republic of Korea

**Keywords:** Diseases, Neurology, Neuroscience

## Abstract

Parkinson’s disease (PD) affects motor and non-motor systems; however, retinal changes and their molecular basis are not well understood. Using a transgenic mouse model overexpressing A53T-mutant human α-synuclein, we examined retinal function, structure, and proteomics at 6- and 16 months. Early retinal dysfunction was detected by a reduction in scotopic oscillatory potential amplitudes on electroretinography. Optical coherence tomography showed early thinning of the retinal nerve fiber layer/ganglion cell layer, and photoreceptor layer, accompanied by thickening of the inner plexiform layer. Phosphorylated α-synuclein accumulation, increased glial fibrillary acidic protein, and loss of the ribbon synapse protein CtBP2 were observed. Proteomic profiling revealed stage-dependent alterations involving α-synuclein, oxidative stress markers, and crystallins. Network analysis showed progression from α-synuclein-associated disruption to inflammation and metabolic remodeling. These results highlight retinal alterations as early indicators of PD neurodegeneration and provide mechanistic insights into the molecular events that precede neuronal loss.

## Introduction

Parkinson’s disease (PD) is a neurodegenerative disorder characterized by the accumulation and degeneration of α-synuclein in the dopaminergic neurons of the central nervous system^1,2^. This pathology leads to neuronal damage and neurotransmitter imbalance, manifesting as both motor and non-motor symptoms. Ophthalmologically, visual impairments such as diplopia, visual hallucinations, and visuospatial defects appear as non-motor symptoms^2^. As an extension of the central nervous system, the retina provides a unique opportunity to study neurodegenerative diseases.

Research on PD has also examined the changes in retinal function and structure. Functional changes in the retina can be assessed through electroretinography (ERG). Previous studies have found reduced photopic b-wave and oscillatory potential (OP) amplitudes in patients with PD^3,4^. This indicates that the visual symptoms of PD may stem from a decline in retinal function, which correlates with the structural changes observed on optical coherence tomography (OCT). Although findings on total macular and overall retinal thickness remain heterogeneous^5,6^, many studies consistently demonstrate thinning of the inner retina, particularly the parafoveal ganglion cell-inner plexiform layer (GCIPL), indicating that selective inner retinal atrophy may represent a reliable structural biomarker in PD^5–10^. Notably, such inner retinal changes can be detected even in the early stages of PD, highlighting the potential of OCT for early diagnosis^5,10–13^. Furthermore, correlations between OCT-derived retinal thinning and disease progression have been reported, supporting its utility for severity assessment^13,14^. The ability to diagnose PD early or assess its severity through relatively simple retinal examinations could substantially enhance patient care and improve quality of life through timely treatment. However, a notable gap exists in the research regarding the molecular or pathological basis underlying these correlations.

This study aimed to use a genetically modified mouse model of PD to investigate structural and functional changes in the retina during the early and late stages of PD. Furthermore, immunohistological and retinal proteomic analyses were performed to better understand the pathophysiology of the retinal changes. These insights could contribute to our understanding of the pathophysiology of PD, ultimately aiding in diagnosis and treatment.

## Results

### α-synuclein accumulation was associated with neuroinflammation remodeling of the inner retinal layers, and progressive disruption of vertical synaptic transmission

**Fig. 1:**
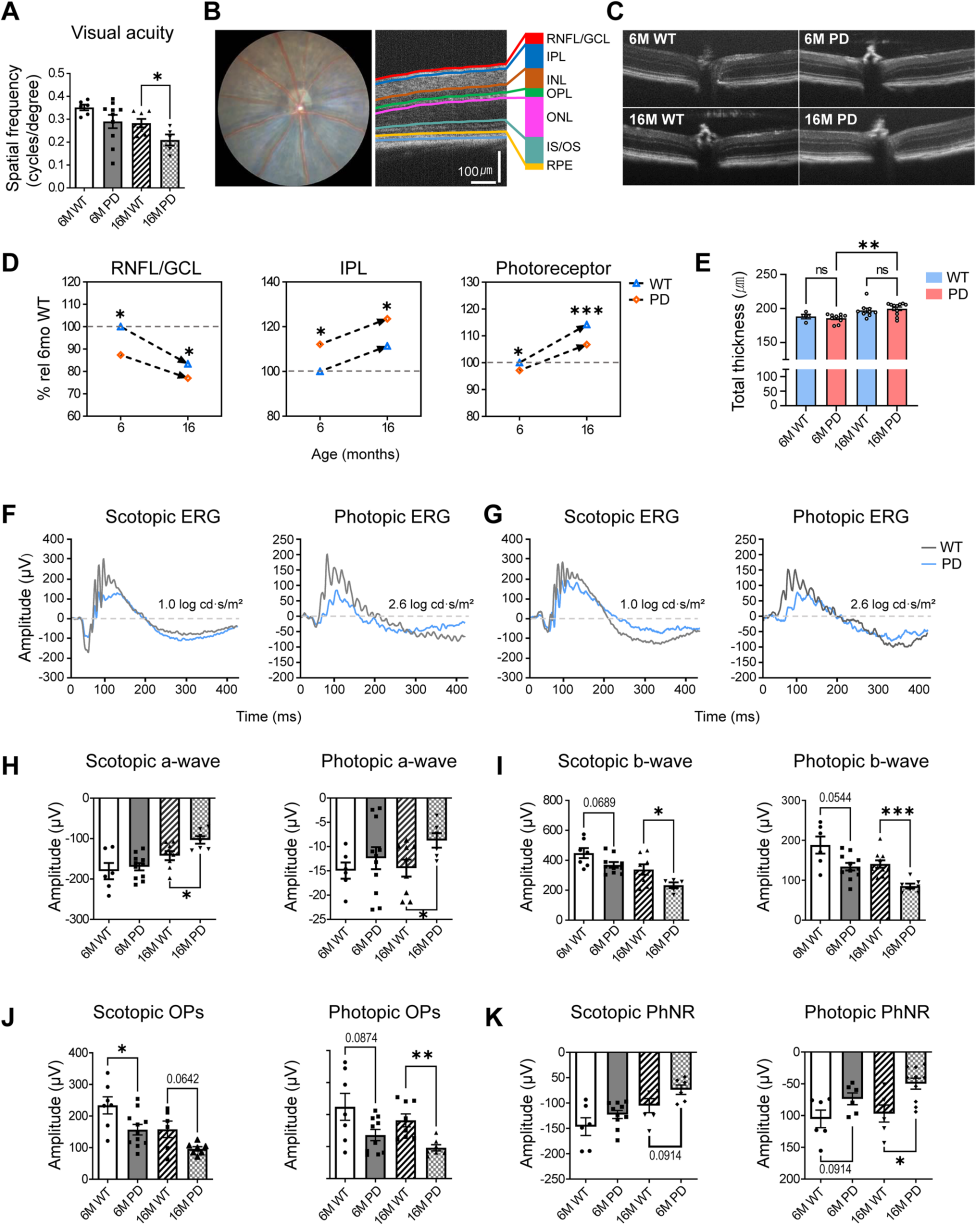
Comprehensive analysis of structural and functional abnormalities in the retina of the M83 Parkinson’s disease mouse model at early and late stages. **A** Visual acuity measured using optokinetic tracking (OKR) showed a reduction in PD mice compared with wild-type (WT) controls, with a significant decline observed at 16 months. **B**, **C** Representative optical coherence tomography (OCT) images showing retinal layer segmentation. RNFL (retinal nerve fiber layer), GCL (ganglion cell layer), IPL (inner plexiform layer), INL (inner nuclear layer), OPL (outer plexiform layer), ONL (outer nuclear layer), and photoreceptor layer (OLM to RPE). **D** Quantitative comparison of retinal layer thickness in PD and WT mice. PD mice exhibited significant thinning of the RNFL/GCL and photoreceptor layers at both 6 and 16 months and increased IPL thickness at both ages, indicating early inner-retinal remodeling. **E** Total retinal thickness measured from the RNFL/GCL to the outer border of the retinal pigment epithelium (RPE). **F**, **G** Representative full-field electroretinogram (ERG) traces recorded under scotopic (1.0 log cd s/m²) and photopic (2.6 log cd s/m²) conditions. PD mice exhibited reduced waveform amplitudes compared with WT controls. (**H**, **I**) Quantification of ERG a- and b-wave amplitudes. At 16 months, both scotopic and photopic responses were significantly reduced in PD mice compared with WT controls, reflecting impaired vertical signal transmission from photoreceptors to bipolar cells. **J** Oscillatory potentials (OPs) recorded under scotopic and photopic conditions. OP amplitude was calculated as the summed peak-to-baseline amplitudes of the first four oscillatory wavelets (OP1–OP4). PD mice exhibited a significant reduction in OP amplitude at 6 months under scotopic conditions and at 16 months under photopic conditions, suggesting early inner-retinal circuit dysfunction followed by cone-driven pathway impairment. **K** Photopic negative response (PhNR) amplitudes representing retinal ganglion cell activity. Photopic PhNR amplitudes were significantly reduced in PD mice at 16 months, whereas scotopic PhNR responses showed no significant differences at either age. Data are presented as mean ± standard error of the mean (SEM). Statistical significance was determined using an unpaired two-tailed Student’s *t*-test (*p<0.05, **p<0.01, ***p<0.001). Scale bars, 100 µm.

OCT and ERG analyses collectively revealed that structural alterations in the inner retina preceded functional impairments of retinal ganglion cells (RGCs) and photoreceptors in PD mice (Fig. 1 and Supplementary Fig. 1).

OCT analysis demonstrated that both the retinal nerve fiver layer/ganglion cell layer (RNFL/GCL) and photoreceptor layers were significantly thinner in PD mice compared to wide-type (WT) controls at 6 months (RNFL/GCL, *p* = 0.0446; photoreceptor, *p* = 0.038) and at 16 months (RNFL/GCL, *p* = 0.0417; photoreceptor, *p* = 0.0007) (Fig. 1D). In contrast, the inner plexiform layer (IPL) was thicker in PD-transgenic (Tg) mice than in WT at both 6 and 16 months and showed an age-related increase in both genotypes (*p* = 0.0189 and 0.0398, respectively), suggesting reactive gliosis or inflammatory remodeling of the synaptic layers. No significant differences were observed in the inner nuclear layer (INL), outer plexiform layer (OPL), outer nuclear layer (ONL), and retinal pigment epithelium (RPE) thickness. These results indicated that α-synuclein accumulation induces early neuroinflammatory remodeling of the inner retina, characterized by IPL thickening and concurrent thinning of the RNFL/GCL and photoreceptor layers.

Functional assessment using ERG and optokinetic testing supported these structural findings. Visual acuity, assessed by optokinetic nystagmus, was comparable between PD and WT mice at 6 months (*p* = 0.0791) but significantly decreased in PD mice by 16 months (*p* = 0.0417) (Fig. 1A). Full-field ERG revealed progressive impairment of vertical signal transmission from photoreceptors to inner retinal neurons (Fig. 1H–K). Scotopic a-wave amplitudes, representing rod-driven responses, declined in both groups with aging but were markedly reduced in 16-month PD mice (*p* = 0.0202). Similarly, photopic a-wave amplitudes, reflecting cone activity, were significantly decreased in PD mice by 16 months (*p* = 0.0279). Although b-wave amplitudes at 6 months did not differ significantly between groups (scotopic: *p* = 0.0689; photopic: *p* = 0.0544), a progressive decline was evident in PD mice, reaching significance at 16 months (scotopic: *p* = 0.0249; photopic: *p* = 0.0002). To further evaluate inner retinal function, OPs and photopic negative responses (PhNRs) were analyzed (Fig. 1J, K). Scotopic OP amplitudes, indicative of amacrine cell activity, were significantly reduced in PD mice at 6 months (*p* = 0.0351) and showed a decreasing trend at 16 months (*p* = 0.0642). Photopic OPs were also significantly decreased in 16-month PD mice (*p* = 0.0034). PhNR amplitudes, reflecting RGC function, were slightly lower in PD mice under scotopic conditions but significantly reduced under photopic conditions at 16 months (*p* = 0.0161).

Collectively, these results demonstrate that α-synuclein accumulation triggers neuroinflammation and structural remodeling of the inner retina, including IPL thickening and RNFL/GCL thinning, which precede measurable deficits in vertical synaptic transmission and progressive visual dysfunction in PD mice.

### α-synuclein aggregation and glial activation precede neuronal alterations in the PD retina

**Fig. 2:**
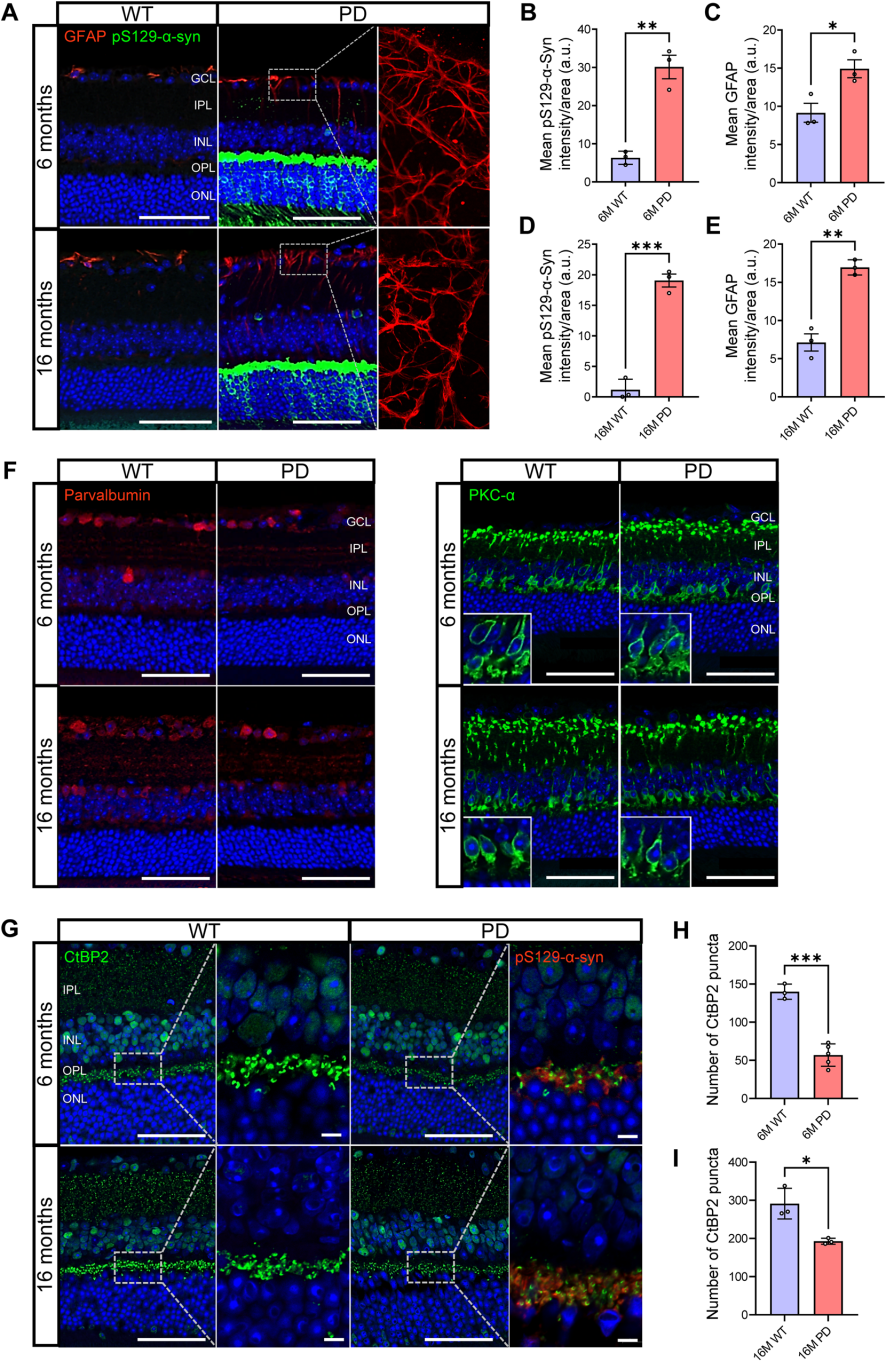
α-Synuclein aggregation and glial activation precede neuronal and synaptic alterations in the PD retina. **A** Immunofluorescence (IF) staining of phosphorylated α-synuclein (pS129-α-Syn) and GFAP in retinal sections from 6-month (6 M) and 16-month (16 M) Parkinson’s disease (PD) transgenic (Tg) mice. PD-Tg mice exhibited markedly increased pS129-α-Syn accumulation accompanied by reactive gliosis, as indicated by elevated GFAP immunoreactivity. **B**, **D** Quantitation of pS129-α-Syn intensity showed significant increases in PD-Tg mice at both 6 M and 16 M. **C**, **E** GFAP intensity was also significantly increased in PD-Tg mice at both ages, indicating persistent Müller cell activation. **F** Representative images of parvalbumin and PKC-α immunoreactivity in WT and PD-Tg retinas at 6 M and 16 M. No significant differences were detected between groups (*p* > 0.05). **G** Representative immunofluorescence images of CtBP2 (green), a ribbon synapse marker, and pS129-α-Syn (red) in the OPL at 6 M and 16 M. **H**, **I** Quantification of CtBP2-positive puncta showed significant reductions in PD-Tg retinas at both 6 M and 16 M, indicating age-progressive loss of ribbon synaptic integrity. Data represent means ± SEM (n = 3-5 per group). *p<0.05, ***p<0.001. Scale bars: 50 µm (overview), 5 µm (enlarged view). GCL ganglion cell layer, IPL inner plexiform layer, INL inner nuclear layer, OPL outer plexiform layer, ONL outer nuclear layer, CtBP2 C-terminal binding protein 2.

Complementing the OCT findings, immunofluorescence analysis revealed early pathological changes in the PD retina (Fig. 2A–E). Phosphorylated α**-**synuclein (pS129-α**-**Syn) was markedly increased in PD mice at both 6- and 16 months, with prominent localization in the OPL. Concurrently, glial fibrillary acidic protein (GFAP) expression was significantly elevated in PD mice at both 6- and 16 months (*p* = 0.0278 and 0.0015, respectively), indicating reactive gliosis involving both astrocytes and Müller cells, consistent with the early retinal neuroinflammation described in neurodegenerative models. At 6 months, GFAP immunoreactivity was largely confined to astrocytic processes in the GCL, reflecting constitutive astrocytic expression. By 16 months, however, vertically oriented GFAP-positive fibers extending through the IPL became evident, indicating Müller cell gliosis under pathological stress. These temporal changes suggest a shift from early astrocytic activation to combined astrocytic and Müller cell gliosis with disease progression.

To assess potential alterations in retinal interneuron populations, parvalbumin and protein kinase C-α (PKC-α)—markers of amacrine and rod bipolar cells, respectively—were examined by immunofluorescence (Fig. 2F). Parvalbumin was predominantly expressed in amacrine cell bodies and processes spanning the INL and IPL, while PKC-α was detected in rod bipolar cells located in the INL, with axonal projections terminating in the IPL. Quantitative analysis of parvalbumin immunofluorescence was performed separately within the RNFL/GCL, IPL, and INL layers (*n* = 5 per group). Parvalbumin labeling, predominantly localized to amacrine cell bodies in the INL and their dendritic arbors in the IPL, did not differ significantly between PD-Tg and WT retinas (*p*>0.05, unpaired two-tailed Student’s *t*-test). Although interindividual variability was observed, no consistent directional change was detected, indicating that subtle circuit-level alterations, if present, would require further validation in larger cohorts. Similarly, PKC-α immunoreactivity, representing rod bipolar cell morphology, showed no significant changes between groups, suggesting no detectable alteration in rod bipolar cell structure within the resolution and sample size of the present analysis.

To further investigate structural correlates of synaptic dysfunction, we analyzed the ribbon synapse marker C-terminal binding protein 2 (CtBP2; RIBEYE), a major component of photoreceptor presynaptic terminals localized within the OPL (Fig. 2G). CtBP2-positive puncta were significantly reduced in both 6- and 16-month PD-Tg retinas compared with age-matched WT controls (*p* = 0.0001 and 0.0141, respectively). This reduction indicates impaired ribbon synaptic integrity emerging as early as 6 months and becoming more pronounced with disease progression. The loss of CtBP2-positive ribbon synapses provides direct structural evidence of outer retinal synaptic disruption, complementing ERG and proteomic signatures of synaptic stress. Together, these findings indicate that α-synuclein accumulation and gliotic remodeling progressively compromise retinal synaptic circuitry.

### Differential expression profiles of retinal proteins in early and late PD

**Fig. 3:**
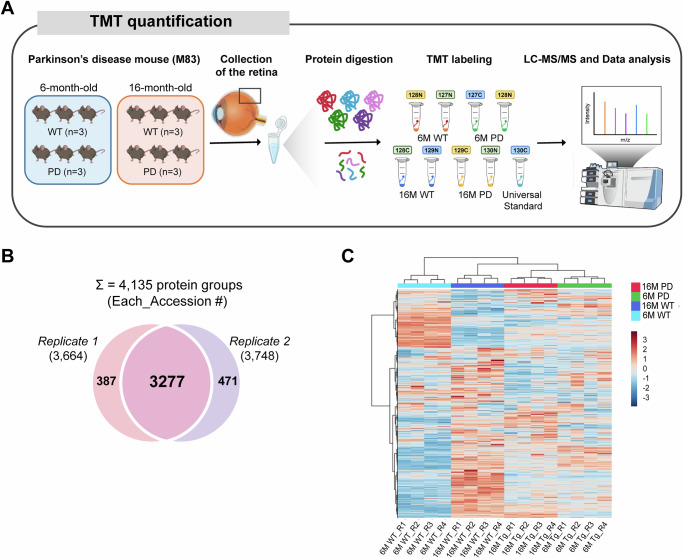
Comprehensive retinal proteome profiling using LC-MS/MS analysis with TMT labeling. **A** A schematic overview of the experimental workflow used for retinal proteome analysis is presented. Retinal tissues from PD and WT mice at 6- and 16 months of age underwent protein extraction, Tandem mass tag (TMT)-based labeling, high-pH reverse-phase fractionation, and subsequent quantitative analysis using liquid chromatography-tandem mass spectrometry (LC-MS/MS). **B** Venn diagram showing the total proteins identified in the retinas of WT and PD mice (*n* = 3/group), with a total of 4,135 protein groups identified. **C** A hierarchical clustering heat map illustrates the quantified proteins, showing clear segregation patterns according to the disease state (PD vs. WT) and time point (6 vs. 16 months). This reflects distinct molecular signatures associated with different experimental conditions. PD Parkinson’s disease, WT wild-type.

To investigate retinal proteomic alterations in Parkinson’s disease, we employed a comprehensive workflow encompassing protein extraction from retinal tissues of PD and WT mice at both 6 and 16 months of age, followed by Tandem mass tag (TMT)-based labeling, high-pH reverse-phase fractionation, and quantitative analysis using liquid chromatography-tandem mass spectrometry (LC-MS/MS) (Fig. 3A). A total of 4135 retinal proteins were identified and quantified with a false discovery rate (FDR) < 1% (Fig. 3B). Unsupervised hierarchical clustering based on the Euclidean distance and average linkage revealed a distinct separation between the PD and WT groups according to genotype and age (Fig. 3C). This pattern suggests that retinal proteomic alterations are more strongly driven by PD-related pathology than by aging alone.

**Fig. 4:**
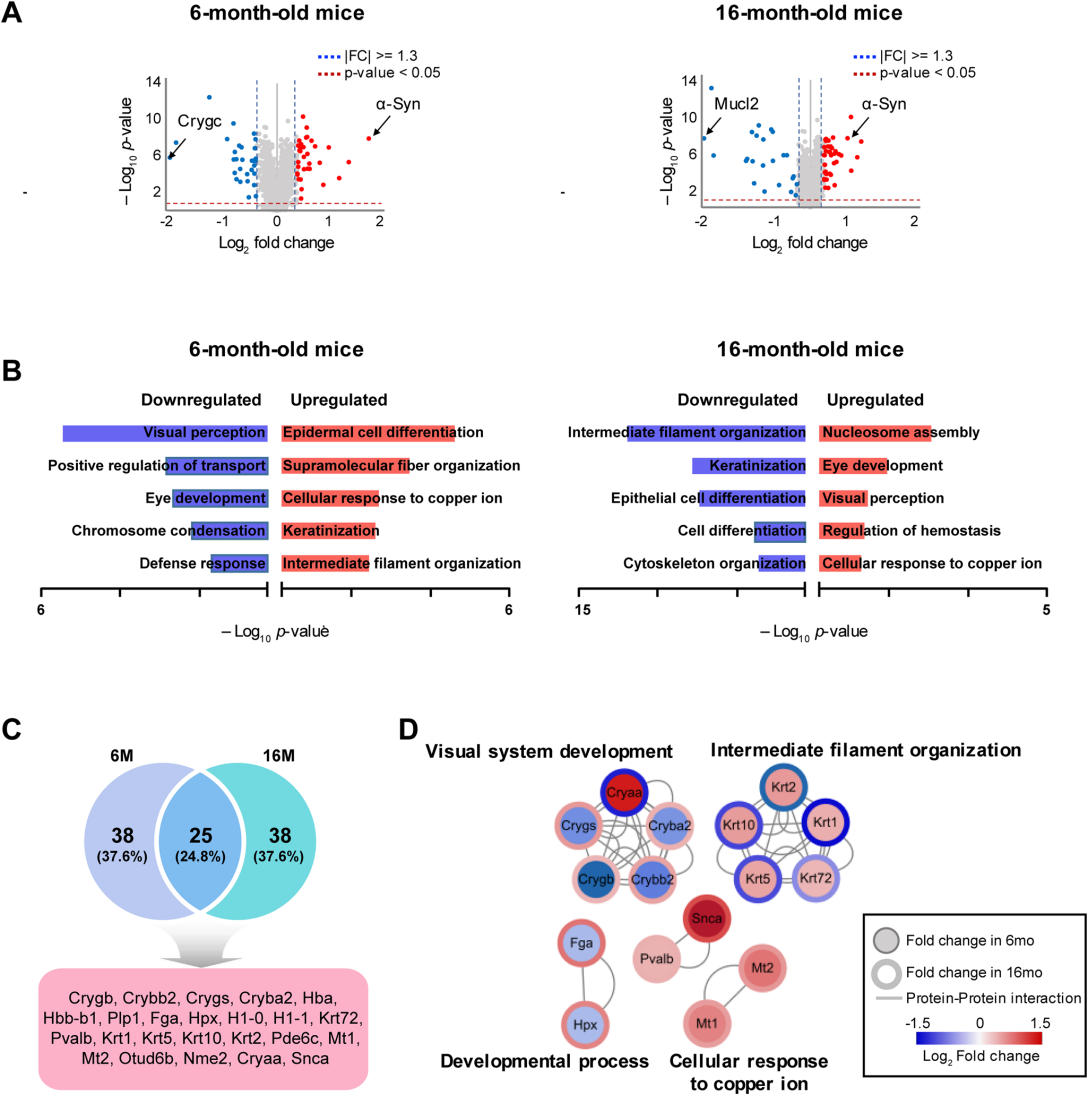
Differentially expressed proteins and functional analysis of the retinal proteome in PD mice. **A** Volcano plot displaying differentially expressed proteins (DEPs) identified through comparative analysis between WT and PD mice retina samples. The vertical dashed lines represent the fold-change threshold ( | FC | ≥1.3), and the horizontal dashed line indicates statistical significance (*p* <0.05). **B** Bar plot showing the top five Gene Ontology Biological Process (GOBP) terms enriched in upregulated and downregulated DEPs. These terms were selected based on statistical significance (*p*-value), providing insight into the key biological pathways most affected in PD. **C** Venn diagram displaying the overlap of differentially expressed proteins between 6- and 16-month-old PD mice. Shared proteins between both time points are indicated, reflecting common molecular alterations across different disease stages. **D** Protein–protein interaction (PPI) network analysis showing the functional relationships between DEPs. Nodes represent individual proteins, while edges depict predicted or known interactions, highlighting key hubs and pathway associations relevant to PD progression. PD Parkinson’s disease, WT wild-type.

Differentially expressed proteins (DEPs) were identified relative to age-matched WT controls, using a threshold of fold-change ≥1.3 and *p* value < 0.05. This threshold was chosen to balance the detection sensitivity and biological relevance, considering that tissue-based proteomic changes are often modest. A volcano plot was generated to visualize the distribution of fold-changes and statistical significance across proteins (Fig. 4A), highlighting a significant increase in the relative abundance of α**-**synuclein (Snca) at both 6- and 16 months in PD mice (*p*<0.001).

Gene Ontology (GO) enrichment analysis using g:Profiler (https://biit.cs.ut.ee/gprofiler/) revealed stage-specific alterations in biological pathways (Fig. 4B). In early PD (6 months), upregulated proteins were primarily associated with epidermal cell differentiation, supramolecular fiber organization, cellular response to copper ions, keratinization, and intermediate filament organization, whereas proteins related to visual perception were downregulated. Notably, proteins related to intermediate filament organization, which were initially upregulated at 6 months, were markedly downregulated at 16 months. In late-stage PD (16 months), the upregulated pathways included nucleosome assembly, eye development, visual perception, regulation of hemostasis, and cellular responses to copper ions (Fig. 4B).

Among the identified DEPs, 25 proteins (24.8%) were commonly dysregulated in both early and late PD (Fig. 4C). Protein-protein interaction (PPI) network analysis using STRING and Cytoscape revealed that the DEPs were enriched in pathways related to visual system development, intermediate filament organization, developmental processes, and oxidative stress responses (Fig. 4D). Consistent with the immunohistochemical findings, Snca exhibited robust upregulation at both disease stages. Similarly, oxidative stress-related proteins, including Pvalb, Mt1, and Mt2, were consistently upregulated, indicating a heightened stress response during disease progression. Proteins such as fibrinogen alpha chain (Fga) and hemopexin (Hpx) were downregulated in early PD, but increased at later stages. Conversely, intermediate filament proteins showed early upregulation, followed by late-stage reduction. Crystallin family proteins also demonstrated dynamic regulation: while Cryaa progressively decreased, Cryba2, Crybb2, Crygb, and Crygs were consistently upregulated with disease progression.

### Protein–protein interaction network topology reveals stage-specific hub proteins involved in retinal remodeling

**Fig. 5:**
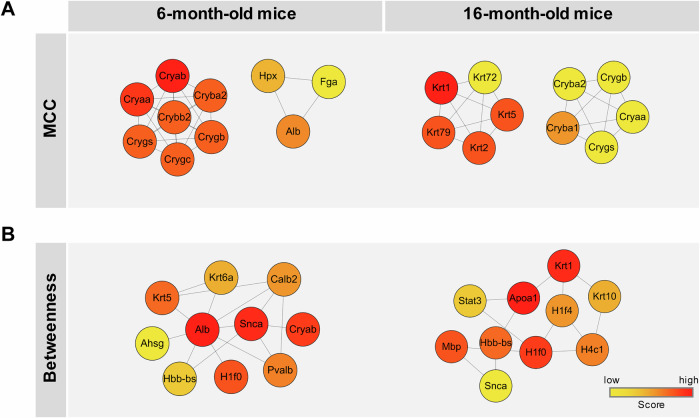
Topology-based network analysis of the top 10 differentially expressed retinal hub proteins in PD mice. **A** Maximal clique centrality (MCC) analysis identifies the top 10 hub proteins in 6- and 16- month-old PD mice. At 6 months, crystallin (Cryaa, Cryab) and acute-phase proteins (Alb, Hpx) exhibit high centrality. At 16 months, crystallins remain prominent, accompanied by keratin family proteins (Krt1, Krt5). **B** Betweenness centrality analysis highlights Snca and Alb as key intermediates in early-stage PD, whereas Apoa1, H1f0, and keratin family proteins gain centrality at 16 months. Node color represents the centrality score (yellow = low, red = high). PD Parkinson’s disease.

To further elucidate the regulatory architecture of retinal protein alterations in PD, we conducted a network topology analysis using maximal clique centrality (MCC) and betweenness centrality (Fig. 5). At 6 months, MCC analysis identified crystallin family proteins (Cryaa, Cryab, and Crybb2) as dominant high-centrality hubs, together with a smaller acute-phase cluster consisting of Alb, Hpx, and Fga. Although Snca did not rank among the top MCC-defined structural hubs, it exhibited high betweenness centrality, indicating that it functions as a key intermediary connecting multiple functional subnetworks during the early stage of disease progression. By 16 months, crystallins remained central in the MCC ranking, but keratin family proteins (Krt1, Krt2, Krt5, and Krt79) also emerged among the top-ranked nodes, reflecting increasing cytoskeletal remodeling with aging and disease progression. In the betweenness analysis, Apoa1 (along with several cytoskeletal/stress-associated nodes) showed higher intermediary roles at 16 months, whereas Snca displayed reduced betweenness over time, indicating a temporal transition from early α**-**synuclein-driven network perturbations to broader inflammatory and metabolic remodeling.

## Discussion

Visual dysfunction is increasingly being acknowledged as a prodromal, non-motor symptom of PD, presenting as diplopia, visual hallucinations, and visuospatial impairment. These impairments are attributed to both central and retinal pathologies, including retinal thinning and disrupted visual processing along central visual pathways^2,12^. Consistent with clinical observations, optokinetic tracking revealed a progressive visual decline in PD mice, supporting this measure as a quantifiable phenotype of PD progression.

To further investigate the retinal basis of this visual dysfunction, ERG was performed, which revealed stage-specific functional impairment across the retinal circuits. At 6 months, a- and b-wave amplitudes were statistically preserved, but a reduction in scotopic OPs was already evident, suggesting early dysfunction of rod-driven amacrine cell circuits. These changes preceded the significant reductions in both scotopic and photopic a- and b-wave amplitudes observed at 16 months, indicating that photoreceptor-driven bipolar cell responses are initially maintained but decline with disease progression. At the late stage, photopic OPs were further reduced and photopic PhNR amplitudes were significantly altered, reflecting progressive cone-driven inner retinal and ganglion cell dysfunction. Interestingly, the temporal pattern of OP impairment differed by light condition: scotopic OPs were significantly reduced at 6 months, whereas photopic OPs declined at 16 months. Together, these findings suggest that rod-driven AII amacrine cell circuits are affected early, followed by progressive involvement of cone-driven amacrine and ganglion cell pathways. This evolving pattern of inner retinal dysfunction supports the notion that early changes arise at the level of synaptic signaling rather than primary neuronal loss, with cone-mediated circuits becoming increasingly vulnerable over time.

These findings are consistent with those of previous clinical ERG studies in PD. Nowacka et al. reported reduced scotopic a-wave, OP, and photopic b-wave amplitudes in patients with PD^3^, whereas Mello et al. observed reductions in photopic b-wave, OP, and PhNR amplitudes^4^, consistent with early dysfunction in bipolar, amacrine, and ganglion cells. More recently, Soto Linan et al. demonstrated reduced photopic b-wave and PhNR amplitudes in both early-stage PD patients and α**-**synuclein transgenic mice, notably showing that female patients exhibited more pronounced deficits in scotopic b-wave and OP amplitudes, highlighting a sex-specific vulnerability and reinforcing the translational relevance of ERG-based biomarkers^15^. This suggests that ERG-detectable functional deficits emerge early in the disease course and are conserved across species.

To our knowledge, longitudinal ERG-based profiling of retinal circuit dysfunction during the progression of PD remains limited. In this study, we performed sequential time-point comparisons at 6 and 16 months to gain insights into the temporal progression of retinal dysfunction. Selective vulnerability of cone-mediated circuits may be partly explained by the role of dopamine in modulating light adaptation and synaptic signaling in amacrine cells, which are known to be compromised in PD^3,16^. Consistently, a study in A53T α-synuclein mice reported preserved numbers of tyrosine-hydroxylase positive amacrine cells despite reduced ERG b-wave amplitudes and thinning of the OPL, and showed that acute L-DOPA administration partially rescued b-wave responses, supporting the notion that early functional impairments may stem from dopaminergic modulation deficits rather than overt neuronal loss^17^. Similarly, Sánchez-Sáez et al. showed that starburst amacrine cells—critical for motion detection—degenerate in PD due to loss of dopaminergic input^18^, underscoring the dopamine-dependent vulnerability of cone-driven inner retinal circuits. However, the differential vulnerability of rod- and cone-driven circuits remains debated. Tran et al. reported greater and earlier dysfunction in cone pathways^19^, whereas Xu et al. demonstrated that α-synuclein aggregates within rod photoreceptor ribbon synapses led to scotopic ERG decline^20^. Ortuno-Lizaran et al. described a marked loss of dopaminergic amacrine cells and their synaptic contacts with AII amacrine cells in PD retinas, providing histological evidence for early scotopic OP deficits^21^. Consistent with this, our analysis revealed reduced CtBP2-positive ribbon synapses in the OPL at both 6 and 16 months, supporting early synaptic vulnerability within the photoreceptor–bipolar cell interface. These discrepancies likely reflect stage- and model-dependent differences, as both rod and cone circuits are ultimately affected.

OCT analysis revealed significant thinning of the RNFL/GCL and photoreceptor layers in PD mice, consistent with previous clinical reports of GC layer atrophy in patients with PD due to degeneration of the GCIPL and INL^7–9,11,22–25^. Recent clinical imaging studies have further emphasized that parafoveal inner retinal and peripapillary RNFL thinning represent some of the earliest structural biomarkers of PD^5^, in close agreement with the RNFL/GCL thinning we observed in our model. Similarly, meta-analytic evidence confirmed significant reduction of peripapillary RNFL, macular ganglion cell complex, and macular thickness in PD patients^6^, supporting the notion that structural alterations of the inner retina are a consistent hallmark across species. However, the clinical literature remains divided on whether total retinal thinning is consistently detected in PD. While a large meta-analysis suggests widespread macular thinning^6^, another report highlights selective vulnerability of inner retinal circuits^5^. Our findings of RNFL/GCL loss without robust total retinal thinning therefore mirror this clinical controversy and suggest that differences in the reported structural alterations of the outer retina across studies may account for these discrepancies. IPL was thicker in PD mice at both ages, not typically reported in patients with PD^7–9,25^, suggesting reactive gliosis or compensatory synaptic remodeling, corroborated by increased GFAP immunoreactivity^19,26^. While INL, ONL, and OPL thicknesses were unchanged, photoreceptor layer thinning preceded late-stage ERG deficits, suggesting early loss or shortening of photoreceptor outer segments rather than somatic layer thinning. A recent OCT analysis in α**-**synucleinopathies demonstrated that INL thinning is one of the robust features distinguishing PD from multiple system atrophy^10^, but the absence of pronounced INL changes in our model may reflect species-specific differences, or stage-specific dynamics.

Immunofluorescence analysis confirmed early molecular changes linked to α**-**synucleinopathy. Phosphorylated α-synuclein (pS129-α-Syn) accumulation was prominent in the OPL as early as 6 months, accompanied by elevated GFAP expression consistent with reactive gliosis. At 6 months, GFAP labeling was largely confined to astrocytic processes in the ganglion cell layer, reflecting basal astrocytic activation; by 16 months, vertically oriented GFAP-positive fibers extended through the IPL, indicating Müller cell gliosis under pathological stress. This temporal shift—early astrocytic activation followed by the emergence of Müller cell gliosis—aligns with progressive neurodegenerative changes. Although GFAP did not meet the predefined threshold for major DEPs, a modest trend toward increased expression was detected in the proteomic dataset, consistent with the gliosis pattern observed by immunohistochemistry. These findings are consistent with human postmortem studies showing that phosphorylated α-synuclein accumulates within retinal ganglion cells and correlates with disease stage and motor severity in PD and incidental Lewy body disease patients^27^, as well as with experimental evidence that rod-specific deletion of VPS35, -a PD-linked gene- induces retinal α-synucleinopathy in mice^28^. Quantitative immunofluorescence of parvalbumin and PKC-α revealed preserved amacrine and rod bipolar cell integrity. Although interindividual variability was observed, overall parvalbumin labeling across RNFL/GCL, IPL, and INL layers remained comparable between PD and WT mice, indicating no consistent alteration within the current sample resolution. Similarly, PKC-α immunoreactivity showed no significant difference, supporting structural preservation of rod bipolar cells at both 6 and 16 months. To further investigate synaptic integrity, CtBP2 analysis revealed a significant reduction in ribbon synapse density within the OPL at both 6 and 16 months, indicating that synaptic impairment emerges early and progresses with disease. The reduction of CtBP2-positive ribbon synapses in PD retinas provides direct structural evidence of outer retinal synaptic disruption, complementing ERG and proteomic signatures of synaptic stress. Together, the data indicate that α-synuclein accumulation and gliotic remodeling contribute to progressive compromise of retinal synaptic circuitry. Although this suggests a PD-associated pathological process, it should be noted that α-synuclein aggregation is not unique to PD; similar retinal deposits have been reported in other proteinopathies such as Alzheimer’s and Huntington’s disease, underscoring the need for cautious interpretation of α-synuclein pathology as a disease-specific biomarker^29^.

Proteomic profiling further reinforced these findings. Unsupervised hierarchical clustering revealed a distinct separation between PD and WT samples at both ages, with genotype exerting a stronger influence than age. Snca was consistently upregulated across both disease stages in PD mice. This proteomic finding is in agreement with our immunohistochemical analysis. It reinforces the central role of α**-**synuclein accumulation as a pathological hallmark of PD and its close association with neurotoxicity, thereby prompting the development of therapeutic strategies targeting α**-**synuclein burden in neurons^30^.

Proteins associated with oxidative stress, such as Pvalb, Mt1, and Mt2, were consistently upregulated across both disease stages in PD. Pvalb, a calcium-binding protein essential for presynaptic calcium signaling and synaptic integration, has shown contrasting results in brain tissue analyses where it is typically downregulated in PD^31,32^. This disparity may reflect the distinct cellular localization and functional roles of Pvalb in the brain and retina, where it is expressed in the cortical GABAergic interneurons and retinal amacrine cells, respectively^32^. Similarly, Pvalb expression is also reported to be upregulated in the peripheral blood from patients with PD, suggesting tissue-specific differences in its regulation^33^. Metallothioneins are metal-binding proteins that play crucial roles in maintaining physiological balance and regulating immune homeostasis^34^. Their upregulation in the retina of PD mice may reflect an adaptive response to oxidative stress in neurodegenerative diseases.

Proteins with dynamic temporal changes in expression were identified. Keratin family proteins associated with intermediate filament organization exhibited early upregulation, followed by late downregulation, indicating transient cytoskeletal compensation that deteriorates under sustained neurodegenerative stress. In contrast, proteins involved in developmental and protective processes, such as Fga, Hpx, and crystallin family members, were downregulated at an early stage but upregulated at 16 months, suggesting delayed activation of regenerative or reprogramming mechanisms in response to neurodegenerative stress. Together, these opposing temporal patterns reflect a shift from early structural compensation to late-stage adaptive remodeling in response to progressive retinal degeneration. Collectively, our findings support a model where pS129-α**-**synuclein accumulation disrupts synaptic integrity at the photoreceptor-bipolar cell interface, leading to early inner retinal dysfunction. This is accompanied by cytoskeletal disorganization and oxidative stress responses, which may further exacerbate functional deficits and precede the structural degeneration of photoreceptors and RGCs.

Topology-based PPI analysis revealed that crystallin proteins such as Cryaa and Cryab remained as central nodes across both disease stages, supporting their conserved role as molecular chaperones that maintain retinal proteostasis under stress conditions^35–37^. At 6 months, a distinct MCC cluster comprising Alb, Hpx, and Fga was observed, whereas at 16 months, keratin nodes emerged among top MCC-ranked proteins, consistent with the systemic inflammatory activation observed in PD and other neurodegenerative diseases. While Snca was not identified as a top hub by MCC, its high betweenness at 6 months points to an early regulatory role in network dynamics, which declined at 16 months when Apoa1 showed a more prominent intermediary position in the betweenness network. These results are consistent with the proteome-level transitions reported in progressive neurodegenerative disease models^38^.

This study has some limitations. First, the relatively small sample size may restrict the generalizability of the findings. Second, species-specific differences in retinal structure and function between mice and humans necessitate caution when translating these results to human PD. Third, the cross-sectional design limited our ability to capture the dynamic changes that occur during disease progression. Finally, although our data suggest roles for α**-**synuclein accumulation and neuroinflammation in retinal pathology, the precise molecular mechanisms remain unclear. Further mechanistic studies are needed to delineate how these factors contribute to visual dysfunction in PD, identify potential retinal biomarkers, and understand the pathophysiological mechanisms.

In conclusion, our findings indicate that early retinal alterations in PD involve synaptic dysfunction, cytoskeletal remodeling, and oxidative stress, which collectively precede overt neurodegeneration. These results highlight the potential of the retina as an accessible and sensitive site for detecting early pathological changes and identifying biomarkers of PD progression. Our study suggests that retinal assessments could facilitate earlier diagnosis and disease monitoring, ultimately enabling timely therapeutic intervention and improving patient outcomes.

## Methods

### Mouse model

Transgenic M83 mice overexpressing the human A53T-mutant form of α**-**synuclein, along with age-matched WT controls, were used in this study. The animals were maintained under specific pathogen-free conditions with a 12-h light/dark cycle and *ad libitum* access to food and water. The experimental groups included 6- and 16-month-old mice. All procedures involving animals were approved by the Institutional Animal Care and Use Committee of Yonsei University (approval number: 2018-0125) and were conducted in accordance with the ARRIVE guidelines and institutional regulations.

### Anesthesia and euthanasia procedures

All in vivo imaging and electrophysiological procedures were performed under general anesthesia. Mice were anesthetized with an intraperitoneal injection of Zoletil (30–40 mg/kg; Virbac, France) and Rompun (xylazine, 5–10 mg/kg; Bayer, Germany) prior to perfusion, tissue collection, and all in vivo imaging procedures (fundus photography, OCT, IOP, and ERG). Adequate anesthetic depth was verified by the absence of pedal withdrawal and corneal reflexes. For animals undergoing perfusion and retinal tissue collection, transcardial perfusion with phosphate-buffered saline (PBS) was performed under deep anesthesia. This procedure was carried out only after confirming a surgical plane of anesthesia, and animals did not regain consciousness at any point. PBS perfusion resulted in cessation of cardiac activity and was considered a terminal procedure in accordance with the AVMA Guidelines for the Euthanasia of Animals (2020).

### Fundus photography

After anesthesia, the pupils were dilated with 0.5% tropicamide and 0.5% phenylephrine mixed eye-drops (Mydrin-P, Santen Pharmaceutical Co, Ltd., Osaka, Japan). The cornea was lubricated with hypromellose 2.5% (Goniovisc^®^, Hub Pharmaceuticals LCC, CA, USA), and the mice were positioned collaterally in front of the fundus camera (Micron® IV; Phoenix Research Labs, Pleasanton, CA, USA), on the right side of a platform that is fixed in front of the lens. We then focused on the retina and obtained the fundus photograph.

### Tonometry

Mice were anesthetized as previously described, and intraocular pressure (IOP) was measured using a rebound tonometer (Icare® TONOLAB tonometer, Colonial Medical Supply, Franconia, NH, USA). IOP measurements were performed in the left eye of mice, according to the manufacturer’s instructions. At least 6 IOP readings were obtained, and average data was used for analysis.

### Optical coherence tomography

Pupil dilation was induced as stated above, and 2.5% hypromellose (Goniovisc®, Hub Pharmaceuticals LCC, CA, USA) was applied to the cornea to maintain hydration. Retinal imaging was performed using a Micron IV OCT system (Phoenix Research Labs, Pleasanton, CA, USA). Mice were positioned consistently on a platform lateral to the right side of the OCT lens. Retinal cross-sections were acquired, and layer thicknesses were quantified using integrated analysis software (Phoenix Research Labs, CA, USA) provided by the manufacturer. To ensure reproducibility, retinal thickness measurements were obtained at 300 μm from the optic nerve head in four quadrants (superior, inferior, nasal, and temporal). The following retinal layers were manually segmented and averaged: RNFL/GCL, IPL, INL, OPL, ONL, and the photoreceptor layer, defined as the region from the outer limiting membrane (OLM) to the inner border of the retinal pigment epithelium (RPE) band (RPE excluded).

### Electroretinogram

ERG recordings were performed using Micron Ganzfeld ERG (Phoenix Research Labs, Pleasanton, CA, USA). Mice were dark-adapted overnight at least 12 h before the experiment for scotopic testing (rod cell response). After anesthesia, the pupils were dilated as previously described. Once the pupil was adequately dilated, we applied hypromellose 2.5% (Goniovisc®) and inserted the electrodes. ERG was recorded with Ganzfeld ERG according to the standard protocol provided with the manual instruments. Scotopic ERGs were obtained in response to increasing flash intensities ranging from −1.7 log cd s/m² to 1.9 log cd s/m². Photopic ERGs were obtained in response to increasing flash intensities ranging from −0.5 log cd s/m² to 4.1 log cd s/m². Ten responses to light stimulation were averaged. The a-wave (as a measure of photoreceptor function), b-wave (as a measure of bipolar cell function), amplitude and implicit times of rod and cone responses were determined. Amplitude and implicit time of the different waves were measured from rod-mediated responses (scotopic b-wave) to light flashes of 1.9 log cd s/m². Cone-mediated responses (photopic b-wave) from light flashes of 4.1 log cd s/m² were recorded. The PhNR was extracted from the same waveforms evoked by 4.1 log cd s/m² flashes. The PhNR was defined as the most negative deflection following the b-wave, measured relative to the pre-stimulus baseline. To minimize noise and enhance measurement reliability a post-b-wave analysis window was selected and within this segment the 10 data points with the lowest amplitude values were identified and averaged to represent the PhNR amplitude. The OPs were isolated from standard ERG recordings by applying a 30–300 Hz band-pass filter. OP amplitudes (OP1–OP4) were measured from baseline to peak, and the sum of the first four OPs was calculated to obtain the total OP amplitude.

### Spatial visual function test

Spatial thresholds for opto-kinetic tracking of sine-wave gratings were measured weekly, using a virtual optokinetic system (OptoMotry, Cerebral Mechanics, Medicine Hat, Alberta, Canada), as previously described. One to three vertical sine-wave gratings moving at 12°/s, drifting either to the left or right, were projected on four surrounding monitors, while an unrestrained mouse stood on an elevated platform in the center of an arena. A video camera was placed on the ceiling of the device, and transmitted the image to the connected computer. Clockwise movement drove the tracking through the left eye, while counterclockwise motion drove it through the right eye. The experimenter judged whether the mouse made or did not make slow tracking movements with its head and body, to follow the drifting grating. Major repositioning of the head and grooming movements was ignored, and the trial was restarted if the presence or absence of tracking was not clear. The maximum spatial frequency capable of driving the head tracking was determined.

### Retinal protein extraction and in-solution digestion

Proteins were extracted from the pooled retinal tissues in equal amounts from three individual retinas combined into a single sample per group. Retinal tissues were first pulverized under liquid nitrogen to ensure complete homogenization and then lysed in 8 M urea and 100 mM ammonium bicarbonate (Cat. #09830; Sigma, USA) with protease inhibitors (Cat. #78442; Thermo Fisher Scientific, Waltham, MA). The tissue powders were further homogenized using a disposable micro-tissue homogenizer, followed by sonication on ice (five pulses of 10 s each, 20% amplitude). After sonication, samples were centrifuged at 14,000 rpm for 15 min to remove debris, and the protein concentration of the resulting supernatant was measured in duplicate using a bicinchoninic acid protein assay (Thermo, USA, Cat. #23235), following the manufacturer’s protocol. For in-solution digestion, 100 µg of total protein per sample was prepared in lysis buffer (8 M urea, 1X protease inhibitor) and incubated at 37 °C for 1 h to facilitate denaturation. Proteins were then reduced by adding 10 mM dithiothreitol (DTT, Cat. #10708984001; Sigma) for 1 h at approximately 25 °C and alkylated with 30 mM iodoacetamide (Cat. #I6125-10G; Sigma) to block the cysteine residues. All reagents were dissolved in 100 mM ammonium bicarbonate. Following reduction and alkylation, trypsin was added at a 50:1 (w/w) protein-to-trypsin ratio, and the samples were incubated overnight at 37 °C for digestion. The reaction was quenched by the addition of 0.4% trifluoroacetic acid (Cat. #28901; TFA, Waltham, MA, USA). After digestion, the peptides were desalted using a C18 Harvard Macrospin Column (Cat. #744101; Harvard, USA). The eluted peptide samples were vacuum-dried and stored at −80 °C until further use.

### Tandem mass tag labeling and high-pH fractionation

Each peptide was reconstituted in 100 mM triethylammonium bicarbonate (TEAB; Cat. #17902, Honeywell, USA) and labeled with TMT 10-Plex isobaric mass-tagging reagents (Thermo, USA, Cat. #90111) following the manufacturer’s instructions. The TMT label agent was dissolved in anhydrous acetonitrile (Sigma Aldrich, USA, Cat. #271004), mixed with equal amounts of peptides, and incubated at room temperature for 1 h for labeling. To quench the reaction, 5% hydroxylamine (Cat. #90115; Thermo Fisher Scientific) in 100 mM TEAB was added. Finally, the chemically tagged samples were pooled into one tube, and peptide separation was carried out by high-pH reverse-phase liquid chromatography fractionation using an Agilent 1260 Series HPLC System (Agilent Technologies, Santa Clara, CA). Peptide fractions were concentrated into 12 pools, dried using a speed vacuum concentrator, and desalted using a C18 spin column.

### Quantitative global profiling and data processing

All the peptide samples were resuspended in 0.1% formic acid and loaded onto an EASY-nLC 1000 system (Thermo Fisher Scientific) coupled with a Q Exactive Plus Mass Spectrometer (Thermo Fisher Scientific). Full mass spectrometry (MS) spectra were acquired at a resolution of 70,000 at *m*/*z* 200 with a scan range of 350–1800 *m*/*z*. The top 15 most intense ions with a normalized collision energy (NCE) of 28% were selected for MS/MS. The dynamic exclusion was set at 30 s.

Raw data were processed using the Proteome Discoverer software (version 1.6.0.16) against the UniProt mouse reference proteome. Trypsin was used as the digestion enzyme, allowing up to two missed cleavages. Carbamidomethylation of cysteine and TMT modifications at the peptide N-terminus and lysine residues were set as static modifications, whereas oxidation of methionine was specified as a variable modification. Peptide and protein FDRs were maintained below 1% using the target-decoy approach. Reporter ion quantification was performed with a 20-ppm mass tolerance. Only the unique peptides were used for quantification. Peptide intensities were normalized using the total sum scaling method to correct for differences in sample loading across TMT channels.

### Immunofluorescence staining

Following euthanasia, eyes were enucleated, fixed in 4% paraformaldehyde at 4 °C for 72 h, and embedded in paraffin. Sections (4 μm) were deparaffinized, rehydrated, and subjected to heat-mediated antigen retrieval using Target Retrieval Solution (Dako, USA, Cat. #S2367). After blocking with 10% normal goat serum (Thermo, USA, Cat. #50062Z) for 1 h, sections were incubated overnight at 4 °C with primary antibodies against phosphorylated α-synuclein (Wako, Japan, Cat. #015-25191) and GFAP (Abcam, UK). After washing, sections were incubated with Alexa Fluor 488-conjugated anti-mouse (Cat. #A11001, Invitrogen, USA) and Alexa Fluor 647-conjugated anti-rabbit secondary antibodies (Jackson ImmunoResearch, USA, Cat. #711-605-152) for 1 h at room temperature. Nuclei were counterstained with DAPI, and the slides were mounted with an antifade mounting solution and covered with coverslips. Immunofluorescent images of the stained sections were captured using an LSM700 microscope (Carl Zeiss, Germany). Image analysis was performed using ImageJ software (National Institutes of Health, Bethesda, MD, USA), in which each color channel was separated and converted into grayscale for further examination. After image adjustment, specific regions of interest (ROIs) were manually selected from each sample. The average fluorescence intensity within these ROIs was determined by subtracting the background fluorescence to eliminate nonspecific signals. The calculated fluorescence intensities were averaged across various independent samples to ensure the accuracy and reproducibility of the results.

### Statistical analysis

To compare functional and structural outcomes between wild-type and transgenic mice at each time point (6 and 16 months), unpaired two-tailed *t*-tests with Welch’s correction were performed using GraphPad Prism (version 10.5.0) to account for unequal variances. Normality and variance homogeneity were not formally tested due to the small and unequal sample sizes. Therefore, Welch’s correction was applied to account for potential heteroscedasticity, which is known to provide greater statistical power than non-parametric alternatives such as the Mann–Whitney *U* test in small-sample conditions. DEPs were defined based on a fold change threshold of 1.3 and a *p* value < 0.05. Heatmap visualization of the protein expression profiles was generated using MetaboAnalyst (https://www.metaboanalyst.ca/). GO enrichment analysis was performed using g:Profiler (https://biit.cs.ut.ee/gprofiler/). PPI networks were constructed using the STRING database (https://string-db.org/) and visualized using Cytoscape (https://cytoscape.org/). A *p* value < 0.05 was considered to indicate statistical significance throughout the study.

## Supplementary information

Supplementary figure 1

## Data Availability

Mass spectrometry proteomic data were deposited in the ProteomeXchange Consortium via the PRIDE partner repository with the dataset identifier PXD066087.
