# Supplementary material for: Early retinal synaptic dysfunction and proteomic remodeling precede neurodegeneration in a Parkinson’s disease model
Source: NPJ Parkinsons Dis. 2026 Jan 15;12:47. doi: 10.1038/s41531-026-01261-7 (PMC12909865; doi:10.1038/s41531-026-01261-7)
Supplement: Supplementary file 1 — Supplementary figure 1 [file 41531_2026_1261_MOESM1_ESM.docx]

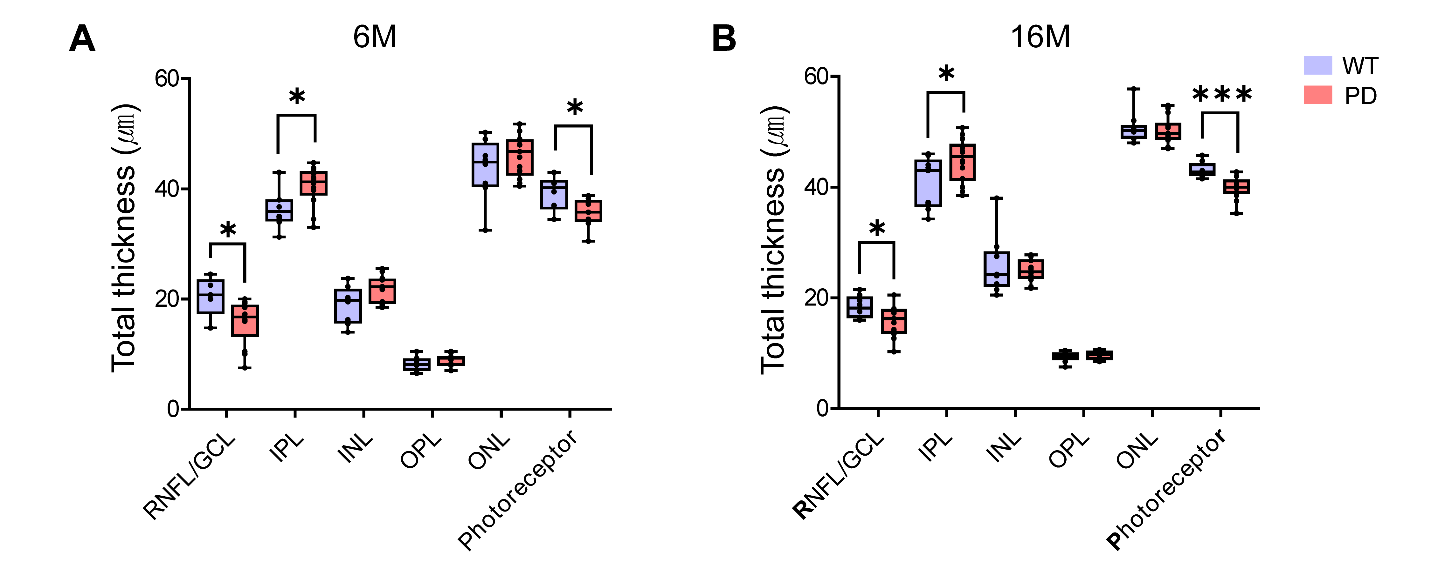


**Supplementary Figure 1. Quantitative analysis of retinal layer thickness in wild-type (WT) and Parkinson’s disease transgenic (PD-Tg) mice using optical coherence tomography (OCT).**

(A-B) Quantitative comparison of individual retinal layers, including retinal nerve fiber layer/ganglion cell layer (RNFL/GCL), inner plexiform layer (IPL), inner nuclear layer (INL), outer plexiform layer (OPL), outer nuclear layer (ONL), and the photoreceptor layer. PD-Tg mice exhibited significant thinning of the RNFL/GCL and photoreceptor layers and increased IPL thickness compared with WT controls at both 6 and 16 months. Data are presented as mean ± SEM (n=5–14 per group). Statistical significance was determined using an unpaired two-tailed Student’s *t*-test with Welch’s correction (**p* < 0.05, ****p* < 0.001).


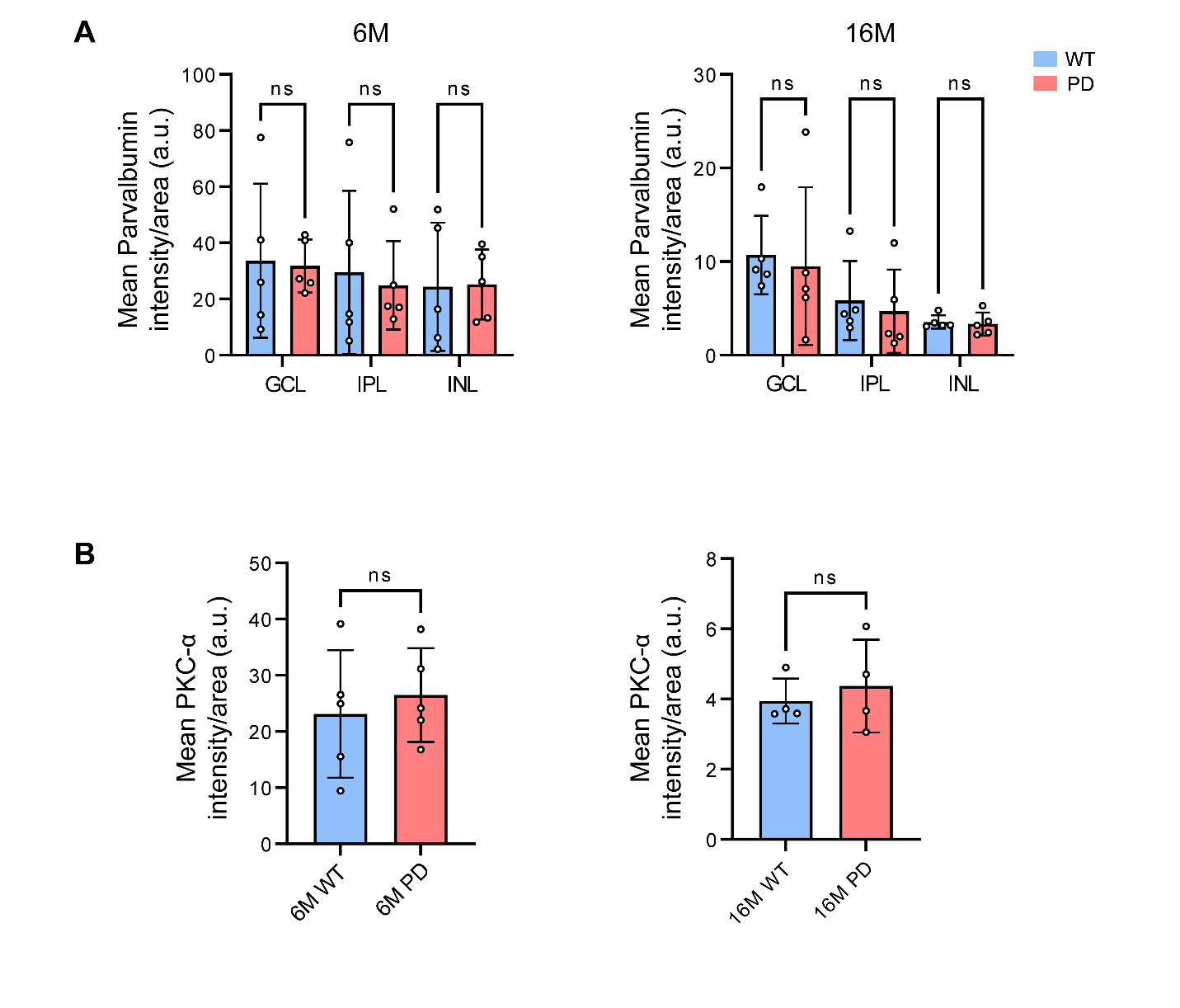


**Supplementary Figure 2. Quantitative analysis of parvalbumin and PKC-α immunofluorescence in WT and PD-Tg (M83) retinas.**

(A) Layer-specific quantification of parvalbumin fluorescence intensity in the GCL, IPL, and INL layers of WT and PD-Tg retinas at 6 and 16 months. (B) Quantification of PKC-α fluorescence intensity in the OPL layer of WT and PD-Tg retinas at 6 and 16 months. Mean fluorescence intensity within each layer was measured using ROI-based analysis from randomly selected retinal regions (n = 5 per group). No statistically significant differences were observed between WT and PD-Tg mice at either age (*ns*, not significant; *p* > 0.05). Statistical analysis was performed using unpaired two-tailed Student’s *t*-test with Welch’s correction. Data are present mean ± SEM.


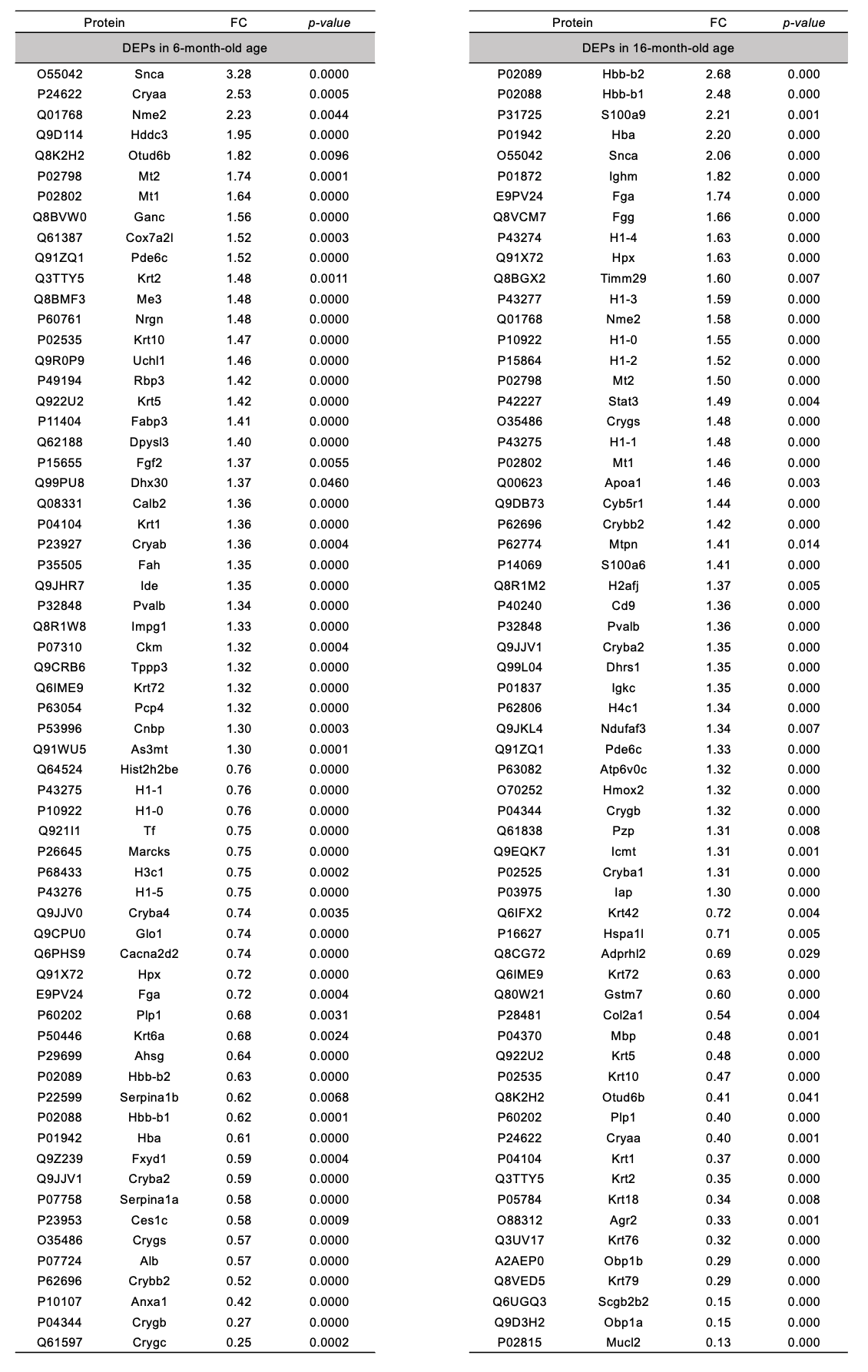


**Supplementary Table 1. List of differentially expressed proteins (DEPs) in the retinas of PD-Tg mice at 6 and 16 months of age compared with WT controls.**
